# Antitumor effects of calgranulin B internalized in human colon cancer cells

**DOI:** 10.18632/oncotarget.7783

**Published:** 2016-02-27

**Authors:** Kun Kim, Kyung-Hee Kim, Kangsan Roh, Byong Chul Yoo, Ja-Lok Ku, Young-Kyoung Shin, Jae Youl Cho, Minjae Kim, Myung-Hee Kwon, Sung Ho Goh, Hee Jin Chang, Jae Hwan Oh

**Affiliations:** ^1^ Colorectal Cancer Branch, Research Institute, National Cancer Center, Goyang, Gyeonggi, Republic of Korea; ^2^ Cancer Research Institute, Seoul National University College of Medicine, Seoul, Republic of Korea; ^3^ Department of Genetic Engineering, Sungkyunkwan University, Suwon, Republic of Korea; ^4^ Department of Biomedical Sciences, Seoul National University College of Medicine, Seoul, Republic of Korea; ^5^ Department of Microbiology, Ajou University School of Medicine, Suwon, Republic of Korea; ^6^ Cancer Genomics Branch, Research Institute, National Cancer Center, Goyang, Gyeonggi, Republic of Korea; ^7^ Center for Colorectal Cancer, National Cancer Center, Goyang, Gyeonggi, Republic of Korea

**Keywords:** calgranulin B, colon cancer, protein internalization, inflammatory microenvironment, aurora A kinase

## Abstract

Calgranulin B is a small, calcium-binding protein expressed in neutrophils that is secreted into the tumor microenvironment in cancer cases. We previously showed that calgranulin B levels are increased in the stools of colorectal cancer patients. In patient tumor tissues, calgranulin B protein levels correlated with the presence of stromal inflammatory cells surrounding tumor cells, and calgranulin B promoter methylation was observed in both paired human tissues and colon cancer cell lines. Cell lines did not express calgranulin B, but *in vitro* studies showed that colon cancer cells internalized extracellular calgranulin B, while other types of cancer cells did not. Calgranulin B internalization led to reduced cell proliferation and increased apoptotic cell death. AKT and ERK signals were also increased after calgranulin B treatment, as were p53, β-catenin, E-cadherin and cleaved caspase-3 levels. Additionally, a human protein microarray identified aurora A kinase as a calgranulin B binding partner, and binding inhibited aurora A kinase activity in a dose-dependent manner. Our findings demonstrate the antitumor effects of calgranulin B in the inflammatory microenvironment and suggest that calgranulin B could be potentially efficacious in the treatment of colon cancer.

## INTRODUCTION

Despite advances in the diagnosis and treatment of colon cancer, its molecular basis is not completely understood. More than 135,000 patients suffer from colorectal cancer annually, which kills about one-fourth of infected individuals and is the most common cause of cancer-related mortality [1, 2]. Calgranulin B, also known as myeloid-related protein 14 (MRP-14) and S100A9, is a small calcium-binding protein expressed in granulocytes, monocytes and activated keratinocytes [3–7]. Calgranulin B has been found in sera from patients with inflammatory diseases including cystic fibrosis, rheumatoid arthritis, systemic lupus erythematosus, Crohn's disease, inflammatory bowel disease and multiple sclerosis [5, 6]. Increased calgranulin B expression has been reported in malignant tissues from patients with colon cancer [8, 9], ovarian carcinoma [10], prostate cancer [11], invasive ductal carcinomas of the breast [12, 13] and lung adenocarcinoma [14, 15], as well as squamous cell carcinoma of the tongue [16].

Calprotectin, a heterodimer of calgranulin A and B, can stimulate β2 integrin Mac 1-mediated fibroblast growth and neutrophil adhesion and act as a neutrophil chemoattractant and macrophage-deactivating factor [17–20]. Calprotectin can also induce signal transduction alterations and cytoskeleton/cell shape changes, and is an important pro-inflammatory mediator in acute and chronic inflammation, as well as in cancer [21, 22]. Calprotectin can induce apoptosis by causing an imbalance between pro- and anti-apoptotic proteins and reactive oxygen species (ROS) [23, 24]. Many reports have also observed calprotectin-mediated apoptosis and cytotoxicity in human leukemia and colon cancer cell lines, as well as in normal cell types including myeloid cells, mitogen-activated lymphocytes, and fibroblasts [25–29]. Calgranulin B is vital to these calprotectin functions; the role of calgranulin B as a component of the calprotectin heterodimer have been well researched, but few studies have explored the role of calgranulin B alone.

Our previous report showed that calgranulin B levels are increased in stools of patients with colorectal cancer [30], but the association between calgranulin B and colon cancer remains unclear. Colon cancer cells activated by calprotectin may increase expression of genes promoting angiogenesis and tumor migration [31]. However, mice lacking calgranulin B showed significantly reduced tumor incidence, growth and metastasis, suggesting that calgranulin B levels, rather than those of the calprotectin heterodimer, correlate with tumor progression. The present study explored the association between calgranulin B and colon cancer cells, and reports the antitumor effect of calgranulin B in the inflammatory microenvironment of colon cancer.

## RESULTS

### Calgranulin B was not expressed in colon cancer cell lines

**Figure 1:**
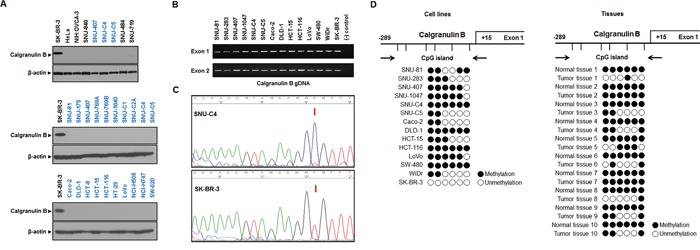
Calgranulin B expression and promoter methylation status in colon cancer cell lines **A.** Calgranulin B was not detected by western blot in colon cancer, gastric cancer, ovarian cancer or cervical cancer cell lines. Only breast cancer cells showed positive calgranulin B protein expression. **B.** gDNA PCR analysis revealed that colon cancer and breast cancer cells carry genes for calgranulin B expression. **C.** Representative DNA sequencing analysis diagrams in SNU-C4 (top, methylated) and SK-BR-3 (bottom, unmethylated) cell lines. **D.** Analysis of calgranulin B promoter CpG island methylation status based on bisulfite sequencing. The distribution of calgranulin B gene CpG dinucleotides is shown, along with DNA sequencing analysis of six promoter CpG dinucleotides. Promoter CpG dinucleotides were mostly methylated in the 13 colon cancer cell lines tested (left panel). Calgranulin B gene methylation was also analyzed in 10 patient tumors with paired normal tissues (right panel). Six promoter CpG islands were completely methylated in all normal tissues, and all tumor tissues showed at least one methylation site. Circle: CpG dinucleotides; closed circle: methylation; open circle: no methylation.

Calgranulin B protein level was analyzed by western blotting in 20 human colon cancer cell lines, two gastric cancer cell lines, one breast cancer cell line, two ovarian cancer cell lines and one cervical cancer cell line. Calgranulin B was not observed in the cancer cell lines tested, excluding the SK-BR-3 breast cancer line (Figure 1A). Genomic DNA (gDNA) PCR performed using SK-BR-3 as a positive control showed that the calgranulin B gene was present in all six colon cancer cell lines randomly selected from 20 cell lines (Figure 1B). Calgranulin B gene promoter methylation status was investigated using methylation-specific PCR after sodium-bisulfite modification. Representative sequence diagrams of the colon cancer cell line SNU-C4 and breast cancer cell line SK-BR-3 are shown (Figure 1C). None of the six CpG sites investigated were methylated in SK-BR-3, but 13 colon cancer cell lines showed at least three methylation sites in the CpG island (Figure 1D, left panel). Calgranulin B promoter regions in all colon cancer patient tissues were also methylated, and hypermethylation was detected in normal tissues (Figure 1D, right panel).

### Calgranulin B levels in tumor tissue were correlated with the presence of stromal inflammatory cells around tumor glands

**Figure 2:**
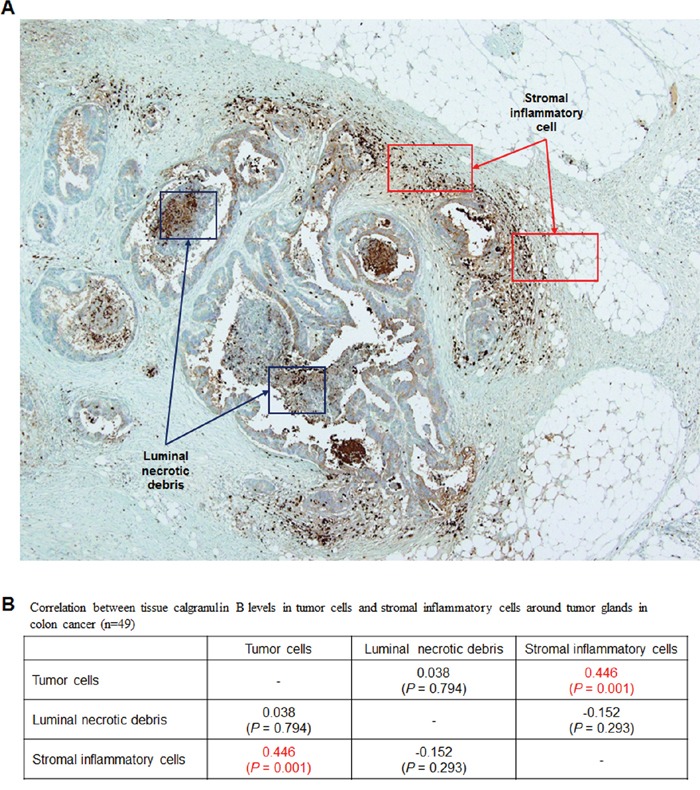
Evaluation of calgranulin B in colon cancer patient tumor tissues **A.** IHC analysis of calgranulin B in patient tissues. Staining was negative in all tumor tissues tested. Most positive calgranulin B staining was observed in tumor cells surrounded by inflammatory cells. **B.** Correlation between tissue calgranulin B levels in colon cancer tumor cells and stromal inflammatory cells around tumor glands. Calgranulin B protein level was estimated in tumor cells, luminal necrotic debris and stromal inflammatory cells (n = 49). Calgranulin B expression in colon cancer tissues was correlated with the presence of stromal inflammatory cells (Pearson correlation coefficient = 0.446, *P* = 0.001).

Calgranulin B was not detected by immunohistochemistry (IHC) in most tumor tissues from 49 colon cancer patients, although some positive calgranulin B staining was observed in inflammatory cells as well as in tumor cells surrounded by inflammatory cells (Figure 2A). Calgranulin B protein levels were analyzed in tumor cells, luminal necrotic debris and stromal inflammatory cells. Calgranulin B expression in colon cancer tissues was correlated with the presence of stromal inflammatory cells (Figure 2B, Pearson correlation coefficient = 0.446, *P* = 0.001).

### Internalization of extracellular calgranulin B into colon cancer cells

**Figure 3:**
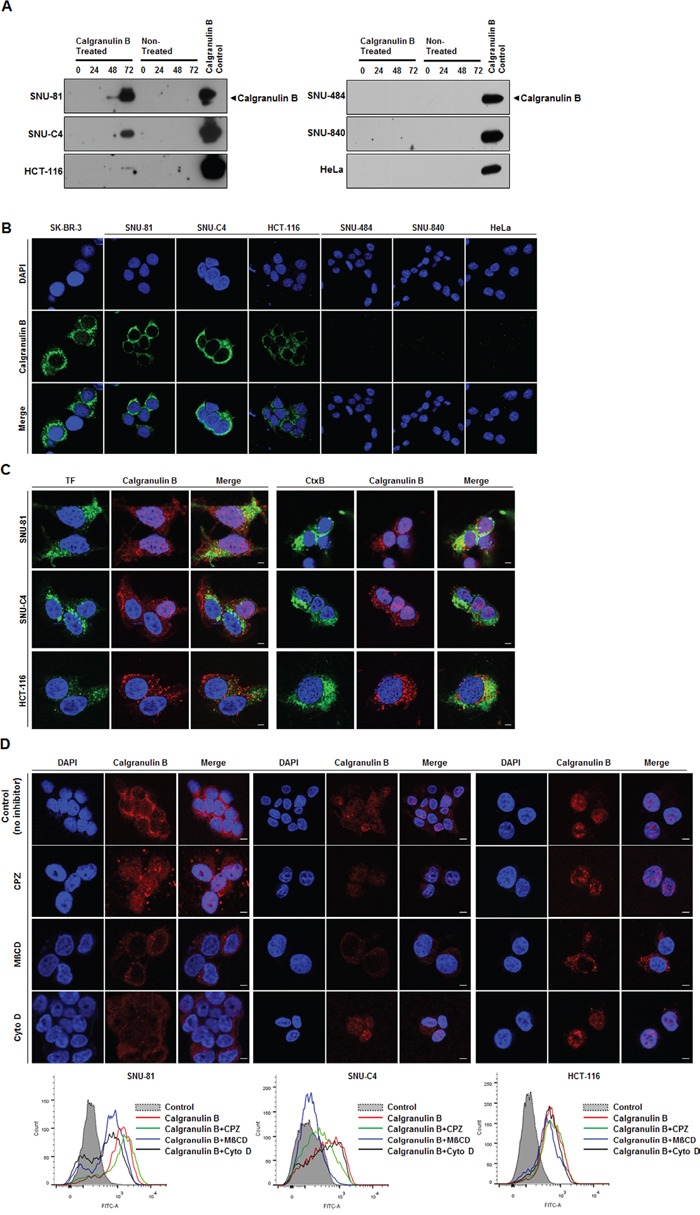
Internalization of extracellular calgranulin B into colon cancer cell lines **A.** Western blot analysis performed after the calgranulin B treatment. Colon cancer cell lines (SNU-81, SNU-C4, HCT-116) had internalized calgranulin B at 72 h post treatment (100 nM calgranulin B), but gastric cancer (SNU-484), ovarian cancer (SNU-840) and cervical cancer (HeLa) cell lines had not. **B.** Confocal microscopy results show internalized calgranulin B in the cytoplasm of colon cancer cells. Nuclei were stained with DAPI. SK-BR-3 was used as a positive control. **C.** Co-localization of calgranulin B with intracellular endocytosis markers. HCT-116, SNU-C4, and SNU-81 cells were co-treated with 100 nM calgranulin B (red) and 10 μg/ml Alexa 488-transferrin (TF, green in the left panel) or 10 μg/ml Alexa 488-cholera toxin-B (CtxB, green in the right panel). At 2 h post treatment, confocal microscopic analysis was performed. Nuclei were visualized via Hoechst 33342 (blue) staining. Scale bars, 5 μm. **D.** Effects of endocytosis inhibitory drugs on calgranulin B uptake in colon cancer cell lines. HCT-116, SNU-C4 and SNU-81 cell lines were incubated with calgranulin B (100 nM) for 2 h with or without pretreatment of CPZ (10 μg/ml), MßCD (5 mM) or and Cyto D (1 μg/ml) for 30 min. Calgranulin B internalization was analyzed using confocal microscopy (upper panel) and flow cytometry (lower panel). Scale bars, 5 μm.

Colon cancer cell lines do not express calgranulin B, but we mimicked the inflammatory cell microenvironment via extracellular treatment with calgranulin B protein (100 nM). Extracellular calgranulin B was absorbed in the cytoplasm of all three colon cancer cell lines tested (SNU-81, HCT-116, SNU-C4), but not others (gastric cancer, SNU-484; ovarian cancer, SNU-840; cervical cancer, HeLa) at 72 h post treatment. Calgranulin B internalization was confirmed by western blot analysis (Figure 3A) and confocal microscopy (Figure 3B). Relatively low uptake of calgranulin B was observed in HCT-116, but was higher in SNU-81 and SNU-C4 (Figure 3A).

To explore the calgranulin B internalization pathway, cells were co-treated with calgranulin B and Alexa 488-labeled transferrin (clathrin-mediated endocytosis, TF), cholera toxin-B (caveolae/lipid raft-mediated endocytosis, Ctx-B) or dextran (micropinocytosis) (Figure 3C). In HCT-116 cells, calgranulin B co-localized with both TF and Ctx-B. Dextran did not enter the three cell lines. Additionally, three inhibitors were used to investigate calgranulin B internalization: CPZ (clathrin-mediated endocytosis), MßCD (caveolae/lipid raft-mediated endocytosis), and Cyto D (macropinocycosis). Confocal microscopy and flow cytometry results showed that internalization was not reduced by the inhibitors in HCT-116 cells (Figure 3D), demonstrating that calgranulin B may enter HCT-116 cells via different endocytosis pathways. Calgranulin B in SNU-C4 cells co-localized with both TF and Ctx-B, and calgranulin B uptake was inhibited by CPZ and MßCD, but not Cyto D. These results suggest that calgranulin B was internalized into SNU-C4 cells by both clathrin-mediated and caveolae/lipid raft-mediated endocytosis. In SNU-81, calgranulin B internalization was inhibited by treatment of MßCD and Cyto D, and it demonstrated that involvement of caveolae/lipid raft-mediated endocytosis and macropinocytosis in the calgranulin B internalization into SNU-81 cells.

### Extracellular treatment of calgranulin B induced antitumor effects in colon cancer cells

**Figure 4:**
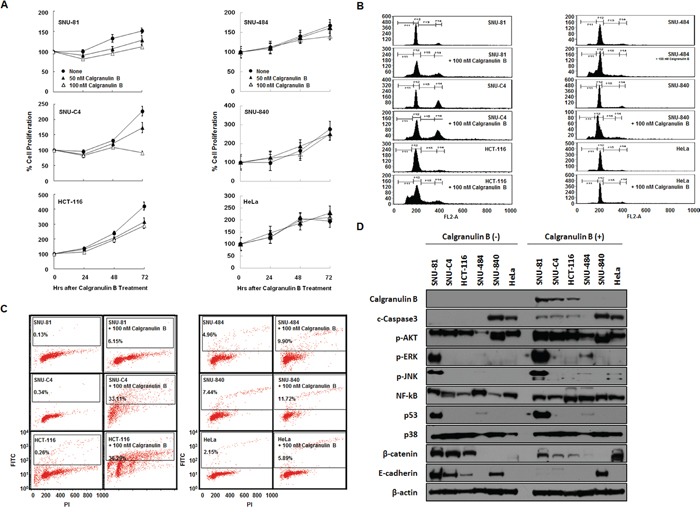
Effects of calgranulin B internalization on colon cancer cell lines **A.** MTT assay results showed increased cell death in SNU-C4 cancer cells 48–72 h after calgranulin B treatment compared to SNU-81 and HCT-116 cells. **B.** FACS analysis confirmed cell cycle changes, most significantly arrest at sub-G1 phase, in all tested cell lines (excluding HeLa) at 72 h post calgranulin B treatment (100 nM). **C.** TUNEL assay showed that apoptosis was effectively increased in colon cancer cell lines at 72 h post treatment. **D.** At 72 h post calgranulin B (100 nM) treatment, intracellular signaling was assessed using western blot analysis. Total levels of cleaved caspase-3 and p53, as well as phosphorylated AKT, ERK and JNK, were increased after treatment. In contrast, β-catenin and E-cadherin levels were suppressed in colon cancer cells.

Extracellular treatment of calgranulin B suppressed proliferation of all three colon cancer cell lines tested, but not others (Figure 4A). However, cell cycle changes were observed in all six cell lines tested following calgranulin B treatment, most significantly arrest at sub-G1 phase (Figure 4B). Apoptotic cell death was induced after 100 nM calgranulin B treatment in colon cancer cell lines compared to others (Figure 4C). Furthermore, extracellular treatment with calgranulin B resulted in intracellular signaling changes in colon cancer cell lines. Total cleaved caspase-3 and p53, as well as phosphorylated AKT, ERK and JNK, were increased following calgranulin B treatment, whereas β-catenin and E-cadherin levels were decreased.

### Calgranulin B bound to aurora A kinase and decreased its activity

**Figure 5:**
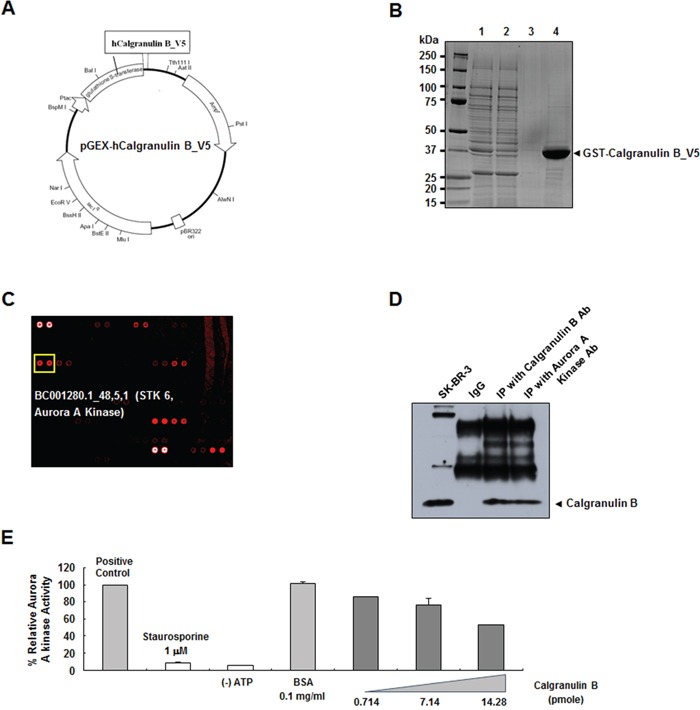
Decreased aurora A kinase activity upon calgranulin B binding **A.** Recombinant human calgranulin B V5-tagged vector construction. The recombinant protein was fused with GST at the N-terminus for purification and the V5 tag at the C-terminus for protein–protein interactions. **B.** SDS-PAGE gel showing the glutathione *S*-transferase (GST)–human calgranulin B fusion protein purified from *E. coli*. Lanes 1–4 indicate whole homogenate, GST column flow-through, GST column wash, and eluted fusion protein, respectively. **C.** In a human protein microarray, aurora A kinase was identified as a calgranulin B binding candidate. **D.** Immunoprecipitation (IP) of calgranulin B and aurora A kinase confirmed the binding. **E.** Calgranulin B binding suppressed aurora A kinase activity in a dose-dependent manner.

Proteins that may bind to calgranulin B were screened using a human protein microarray. Expression vector for GST-human calgranulin B fusion protein with a V5 tag was constructed (Figure 5A). The fusion protein (∼37 kDa) expressed in *E. coli* was purified (Figure 5B), and used as a probe in microarrays with approximately 4,000 proteins. Candidates with Z-scores < 3 were removed, and 71 proteins were found to bind calgranulin B (Supplementary Data 1). Aurora A kinase appeared to strongly bind calgranulin B (Figure 5C) and this interaction was confirmed via reverse immunoprecipitation (Figure 5D). In the presence of calgranulin B, aurora A kinase activity decreased in a dose-dependent manner (Figure 5E). Enzyme activity decreased by approximately 13% more than the positive control in the presence of 200 ng/ml (14.28 pmol) calgranulin B (Figure 5E).

## DISCUSSION

Calgranulin B was originally discovered as an immunogenic protein expressed and secreted by inflammatory cells [32]. Calgranulin B expression has since been observed in myeloid cells, lymphoid cells, cancer cells and tumor stromal cells [33]. In particular, increased calgranulin B levels have been reported in numerous cancer types including colon cancer, gastric cancer, breast cancer, hepatocellular carcinoma and non-small cell lung cancer [34–47]. Similar to other types of cancer, several previous studies showed elevated calgranulin B levels in serum, tissue and stool of colon cancer patients [30, 37, 48, 49]. Calgranulin B expression in poorly differentiated colon cancer tissues was higher than that in well- and moderately differentiated tissues, and levels in advanced colon cancer samples were much higher than in early stage samples [49]. Calgranulin B expression in tumor cells was thus associated with histological grade, Dukes stage and lymph node metastasis [49].

Recent studies support a pathological role for calgranulin B in colon cancer. While our data clearly show that colon cancer cell lines did not produce calgranulin B protein (Figure 1A), in IHC studies, calgranulin B in colon cancer tissues was correlated with the presence of stromal inflammatory cells (*P* = 0.001; Figure 2). Our findings suggested that calgranulin B is not expressed in colon tumor cells and that calgranulin B in these cells may result from the uptake of calgranulin B secreted by inflammatory cells in cancer stromal tissue.

Extracellular calgranulin B was internalized in colon cancer cell lines *in vitro*, but this was not observed in other cancer cell lines (Figure 3). To address the ability of colon cancer cells, but not other types of cancer cells tested, to internalize calgranulin B present in culture medium, the effects of three endocytosis inhibitors (CPZ for clathrin-mediated endocytosis; MßCD, caveolae/lipid raft-mediated endocytosis; Cyto D, macropinocycosis) were investigated (Figure 3D). None of the inhibitors blocked calgranulin B uptake by the colon cancer cell lines. We concluded that calgranulin B entered colon cancer cell lines via an alternative endocytosis pathway, although our results did not allow us to define the specific pathway.

Colon cancer cell lines exhibited cell cycle arrest, apoptotic cell death and decreased cell proliferation rates following calgranulin B uptake (Figure 4). Extracellular calprotectin has growth-inhibitory properties and promotes cytotoxicity and apoptosis in many different human and mouse tumor cell types [50]. Calprotectin expression in cancer cells has been associated with tumor development, cancer invasion and metastasis [50]. However, a recent study suggests that calgranulin B can promote or inhibit tumor growth in cancer depending on the molecular environment [33, 51]. Calgranulin B seems to inhibit cancers at higher concentrations and may promote tumor growth at lower concentrations [51]. The present study showed that calgranulin B may suppress colon cancer cell proliferation (Figure 4), but this does not address the effects of the calgranulin A-B complex.

Calprotectin has been reported as an endogenous TLR4 agonist, leading to activation of NF-κB [52]. In the tumor microenvironment, calprotectin secreted by myeloid cells binds to RAGE on tumor cells in a carboxylated-glycan-dependent manner, promoting activation of MAPK signaling pathways and NF-κB [51]. Increased calgranulin B may promote apoptosis via both p53-dependent and -independent pathways [31]. The present study showed enhanced AKT and ERK signaling and increased p53 protein levels after treatment of SNU-81 colon cancer cells with extracellular calgranulin B (Figure 4D). Calgranulin B treatment commonly increased AKT phosphorylation and decreased β-catenin and E-cadherin, but increased NF-kB signaling was only observed in HCT-116 cells (Figure 4D). Cleaved caspase-3 also increased after calgranulin B treatment, indicative of apoptotic cell death. However, most calgranulin B-induced signaling changes were favorable for tumor progression, suggesting that decreased β-catenin expression is important for calgranulin B antitumor effects.

To clarify the antitumor function(s) of internalized calgranulin B, we performed a human protein microarray and identified aurora A kinase as a calgranulin B binding partner (Figure 5, Supplementary Data 1). Aurora A kinase is required for centrosome maturation, and centrosomal anomalies have been demonstrated during tumor formation and progression [53]. Aurora A kinase overexpression, reported in malignancies such as colon and gastric cancers [54–56], inhibits p53 family members and suppresses apoptosis and cell cycle arrest [57]. Several aurora kinase inhibitors have been developed as anticancer drugs (AZD1152, MLN8054, MLN8237) and are currently at the preclinical or clinical stages [57]. We found that calgranulin binding inhibited aurora A kinase activity, suggesting a possible mechanism for the observed calgranulin B antitumor effects in colon cancer.

In conclusion, we found that calgranulin B internalized specifically into colon cancer cells; other types of cancers (excluding breast cancer) did not express calgranulin B or take up extracellular calgranulin B protein. Calgranulin B internalization induced apoptosis signaling and reduced cell proliferation, possibly through binding and inhibition of aurora A kinase. Our results suggest that calgranulin B could be potentially efficacious in the treatment of colon cancer.

## MATERIALS AND METHODS

### Human cancer cell lines

Human cancer cell lines were obtained from the Korean Cell Line Bank (KCLB, Seoul, Korea). Colon cancer cell lines: SNU-81, SNU-175, SNU-283, SNU-407, SNU-769A, SNU-769B, SNU-1040, SNU-1047, SNU-C1, SNU-C2A, SNU-C4, SNU-C5, Caco-2, DLD-1, HCT-8, HCT-15, HCT-116, HT-29, LoVo, NCI-H508, NCI-H741, SW-480, SW-620, WiDr; breast cancer cell line: SK-BR-3; gastric cancer cell lines: SNU-484, SNU-719; ovarian cancer cell lines: SNU-840, NIH:OVCA-3; cervical cancer cell line: HeLa.

### Western blot analysis

Western blot analysis was performed as described previously [30]. Briefly, protein samples were subjected to sodium dodecyl sulfate–polyacrylamide gel electrophoresis (SDS-PAGE), transferred to polyvinylidene fluoride (PVDF) membranes (Millipore, Bedford, MA, USA) and blocked in 5% non-fat dry milk for 1 h at room temperature. Membranes were incubated with primary antibody against calgranulin B (Santa Cruz Biotechnology, Dallas, TX, USA), aurora A kinase (Abcam, Cambridge, MA, USA), c-Caspase3 (Cell signaling, Massachusetts, USA), p-AKT (Cell signaling), p-ERK (Cell signaling), p-JNK (Cell signaling), NF-κB (Cell signaling), p53 (Cell signaling), p38 (ABcam), β-catenin (ABcam), E-cadherin (Cell signaling), and β-actin (Santa Cruz Biotechnology) or actin (Sigma-Aldrich, St. Louis, MO, USA). Membranes were washed and incubated with horseradish peroxidase (HRP)-conjugated secondary antibody (Southern Biotech, Birmingham, AL, USA). Finally, membranes were rewashed (3 × 15 min), incubated with WEST-ZOL^®^ chemiluminescence reagent (iNtRON Biotechnology, Seoul, Korea) for 1 min and exposed to film (Blue XB-1, Kodak, Rochester, NY, USA).

### gDNA PCR

Fragments encompassing the entire calgranulin B gene were amplified by polymerase chain reaction (PCR) in a total reaction volume of 15 μl containing 100 ng of genomic DNA, 10 pmol of each primer, 250 μl each dNTP, 0.5 units of Taq polymerase, and reaction buffer provided by the supplier (Qiagen, Hilden, Germany). Samples were denatured for 5 min at 94°C in a GeneAmp PCR system 9700 (Applied Biosystems, Foster City, CA, USA), followed by 35 cycles of 94°C for 30 s, 54°C (exon 1) or 57°C (exon 2) for 30 s, and 72°C for 1 min, with a final 7 min elongation at 72°C. PCR was performed using the following primers for calgranulin B: exon 1, sense 5′-TCCCAACTCTTGGTTTTCCA-3′ and antisense 5′-CAGGAGCTCAAATCTTCCCC-3′; exon 2, sense 5′-AGGGTGAGGCTTCCCTTGTA-3′ and antisense 5′-TCCTGATTAGTGGCTGTGGC-3′.

### Bisulfite-modified sequencing analysis

Genomic DNA from cell lines was isolated using the cell culture DNA kit (Qiagen) and subjected to additional proteinase K treatment. Bisulfite treatment was performed using the EZ DNA MethylationTM Kit (Zymo Research, Orange, CA, USA) and bisulfite sequencing primers designed as described previously [58]. The bisulfite sequencing primers used were as follows: calgranulin B primer sense 5′-TGAGTGGTGTTAGAGGAGTAGT-3′ and antisense 5′-CCACACAAAATATTTACCAAAACTATAC-3′. Sequencing of the PCR product was performed in a total reaction volume of 50 μl containing 150 μM dNTPs, 0.3 μM each primer, 1× PCR buffer (Qiagen), Platinum^®^ Taq DNA Polymerase (Invitrogen, Carlsbad, CA, USA) and 2 μl (40 ng) of converted DNA. Denaturation at 94°C for 2 min was followed by 40 cycles of 95°C for 30 s, 54°C for 30 s, and 72°C for 45 s, with a final 7 min elongation at 72°C. PCR products were verified by agarose gel electrophoresis, and sequences were analyzed using a Taq dideoxy terminator cycle sequencing kit on an ABI 3730 DNA Sequencer (Applied Biosystems).

### Immunohistochemistry

IHC was performed as described previously [59, 60]. The study population consisted of 49 colorectal cancer tissues surgically resected at the National Cancer Center, Korea. All tissue samples were obtained with written informed consent. All cases were adenocarcinomas, classified according to World Health Organization (WHO) criteria and staged according to the criteria of the International Union Against Cancer. Tissues were routinely fixed in 10% buffered formalin, embedded in paraffin blocks and sectioned to 4 μm. Immunostaining was performed using the labeled streptavidin–biotin complex (LSAB) method, and primary calgranulin B antibody (1:400; clone H-90, Santa Cruz Biotechnology) was applied after antigen retrieval. Reaction products were not present when nonimmune serum or phosphate-buffered saline (PBS) was used instead of primary antibodies. Calgranulin B cytoplasmic expression was considered positive and results were evaluated semi-quantitatively using a double scoring system that evaluated both staining intensity and percentage of stained cells. Staining intensity was classified as follows: 1, weak; 2, moderate; 3, strong. Percentages of stained tumor cells were assigned the following scores: 0, <10%; 1, 10–25%; 2, 26–50%; 3, >50%. Multiplying the staining intensity by the staining percentage score gave an immunoreactivity score from 0 to 9. Immunoreactivity scores for tumor cells, stromal inflammatory cells and luminal necrotic debris were evaluated separately. Correlation coefficients were estimated using the Pearson correlation method.

### Confocal microscopy

Cells were seeded in a 24-well plate (1 × 10^5^ cells/well) over glass coverslips the day before use and treated with calgranulin B (100 nM) for 2 h at 37°C. After washing with PBS, cells were fixed with 4% paraformaldehyde (PFA) in PBS for 10 min at RT and then permeabilized with Perm buffer [1% bovine serum albumin (BSA), 0.1% saponine, 0.1% sodium azide in PBS] for 10 min at RT. Cells were then incubated with rabbit anti-calgranulin B IgG (1:100; Santa Cruz Biotechnology), followed by Alexa 647-goat anti-rabbit IgG (1:200; Invitrogen). Nuclei were stained with Hoechst 33342 during the last 10 min of incubation at RT. In endocytosis marker tests, cells were co-treated with calgranulin B (100 nM) and either Alexa 488-transferrin (10 μg/ml), Alexa 488-Ctx-B (10 μg/ml) or Alexa 488-dextran (10 μg/ml) in TOM™ Transfection Optimized Medium (WelGENE, Daegu, Korea) for 2 h at 37°C prior to cell fixation and permeabilization. For the endocytic pathway inhibition tests, cells were incubated with calgranulin B (100 nM) for 2 h at 37°C with pretreatment of chloropromazine (10 μg/ml), MßCD (5 mM) or cytochalasin D (1 μg/ml) for 30 min at 37°C, followed by cell fixation and permeabilization.

### Flow cytometry

Flow cytometry was performed as described previously [61]. Cells were seeded at a density of 1 × 10^5^ cells/well and incubated in TOM™ Transfection Optimized Medium (WelGENE) for 30 min at 37°C. Preincubated cells were treated with each inhibitor for 30 min at 37°C before calgranulin B (100 nM) treatment. Cells suspended with trypsin were treated once more with 0.1% trypsin for 3 min at 37°C to wash off the surface-bound protein. After one wash with ice-cold PBS, cells were fixed and permeabilized. Cells were washed with ice-cold PBS twice, labeled with rabbit anti-calgranulin B IgG (1:200; Santa Cruz Biotechnology) followed by fluorescein-donkey anti-rabbit IgG (1:200; Millipore), and then analyzed using FACSCanto II (BD Biosciences, Franklin Lakes, NJ, USA). For each test, 1 × 10^5^ cells were analyzed.

### MTT assay

A colorimetric assay using the tetrazolium salt 3-[4,5-Dimethylthiazol-2-yl]-2,5-diphenyltetrazolium bromide (MTT) was used to monitor cell proliferation suppressed by treatment of calgranulin B. Briefly, cells were seeded into a 96-well plate in 0.18 ml/well culture medium with 0.02 ml of calgranulin B (OriGene, Rockville, MD, USA) or PBS (for untreated control taken as 100% survival). After 4 days, 0.1 mg MTT was added to each well and incubated at 37°C for 4 h. Plates were centrifuged at 450 × g for 5 min at RT, after which the medium was removed. Dimethyl sulfoxide (DMSO; 0.15 ml) was added to each well to solubilize crystals and plates were immediately read at 540 nm using a scanning multiwell spectrometer (Molecular Devices, Sunnyvale, CA, USA). All experiments were performed three times, and IC_50_ (μg/ml) was presented as mean values ± SD.

### Cell cycle analysis

Changes in cell cycle were assessed using a FACSCalibur flow cytometer (Becton Dickinson, San Jose, CA, USA) and CellQuest software (Becton Dickinson). Propidium iodide (PI)-positive cells were quantified as a percentage.

### TUNEL assay

Terminal transferase dUTP nick end labeling (TUNEL) assay was performed with FITC-anti-BrdU staining using an APO-BRDU Kit (Phoenix Flow Systems, Phoenix, AZ, USA). Briefly, 1 × 10^6^ cells were fixed in 1% PFA with PBS. Then, 5 ml of 70% ethanol was added and incubated for 20 h at −20°C. Cells were centrifuged, washed, suspended in a DNA labeling solution and incubated for 1 h at 37°C. Cells were then incubated with FITC-anti-BrdU antibody for 30 min, incubated for 15 min with 0.5 ml of a PI/RNase A staining solution, and analyzed using a FACSCalibur flow cytometry system (Becton Dickinson).

### Construction of the glutathione S-transferase (GST)–human calgranulin B fusion protein

Total RNA was extracted using TRIzol reagent (Invitrogen) and was used to synthesize double-stranded cDNA with a T7-(dT) 24 primer and Superscript II Reverse Transcriptase (Invitrogen). Full-length human calgranulin B was prepared using nested PCR with the 5′ end primer (outer: 5′-GCTTTGGGACAGAGTGCAAG-3′, inner: 5′-ACGGATCCACCATGACTTGCAAAATGTCGCAGC-3′, to add a *BamH*I site one base upstream from the start codon, ATG) and 3′ end primer (outer: 5′-CCACAGCCAAGACAGTTTGA-3′, inner: 5′-TTAAGCTTACGTAGAATCGAGACCGAGGAGAGGGTTAGGGATAGGCTTACCGGGGGTGCCCTCCCCGA-3′, to add the V5 tag). PCR products were ligated into the *BamH*I and *Hind*III sites of the pGEX-2T plasmid to generate recombinant fusion proteins with GST at the N-terminus and the V5 tag at the C-terminus. Transformed *Escherichia coli* cells were grown in Luria–Bertani (LB) broth containing 50 mg/ml of ampicillin at 37°C overnight. Cultures were diluted 100-fold in fresh LB plus ampicillin, and incubation was continued at 37°C. For growth curve determination, samples were taken every 1 h and optical density was measured at 600 nm until the mid-log phase. Isopropyl β-D-thiogalactopyranoside (IPTG) was added to a final concentration of 1 mM, with incubation at 37°C for 6 h. Cells were then homogenized in PBS (pH 8.0), and whole homogenates were centrifuged at 10,000 × g for 20 min. The supernatant containing cytoplasmic extracts was subjected to chromatography on a Glutathione Sepharose 4 Fast Flow column (GE Healthcare Life Sciences), and GST-Calgranulin B fusion proteins were eluted using 50 mM Tris–HCl (pH 8.0) with 10 mM reduced glutathione.

### Human protein array

The ProtoArray™ Kit (Invitrogen) was used to detect protein-protein interactions following the manufacturer's instructions. Briefly, The Human Protein Microarray nc v2.0 was placed in an incubation chamber/hybridization chamber (included in the ProtoArray™ kits, Invitrogen) and 20 ml freshly prepared PBST blocking buffer applied to the side of the chamber. GST-V5-tagged calgranulin B protein (50 μg/ml) was used as a probe. The array was incubated for 1.5 h at 4°C without shaking. Anti-V5 Alexa Fluor^®^ 647 (20 ml) was prepared in fresh PBS Probing Buffer (0.1 μg/ml) and used for incubation with gentle agitation at 4°C for 30 min. The chamber containing the array was inverted briefly on an absorbent surface to completely decant each wash without allowing the array to dry. The protein array was subsequently scanned with the GenePix^®^ 4000B Fluorescent Scanner (Axon Instruments, Foster City, CA, USA). Data were acquired with GenePix^®^ Pro software (Axon Instruments) and processed using a ProtoArray™ Prospector (Invitrogen). The Z-score reflecting a protein binding specificity was calculated as follows: *Z_i_ = (X_i_ –μ_s_)/σ_s_*, where *X_i_* is the signal intensity value of the *i*^th^ protein, and *μ_s_* and *σ_s_* are the mean and the standard deviation of signal intensity of all proteins, respectively.

### Immunoprecipitation

Immunoprecipitation was performed as described previously at 4°C, unless otherwise specified [62]. Approximately 10^7^ cells in 1 ml of cold 1× RIPA buffer containing protease inhibitors (Roche Diagnostics, Basel, Switzerland) were incubated on ice for 30 min with occasional mixing. Cell lysates were centrifuged at 12,000 × g for 10 min. The supernatant was collected, mixed with primary antibody [calgranulin B (Santa Cruz Biotechnology) or aurora A kinase (Abcam)], and incubated for 2 h with rocking. Prepared protein G Sepharose beads (100 μl; GE Healthcare Life Sciences) were added, incubated on ice for 1 h with rocking and centrifuged at 10,000 × g for 30 s. The supernatant was removed and Protein G Sepharose beads were washed five times with 1 ml of cold 1× RIPA to minimize background. Next, 100 μl of 2× SDS sample buffer was added to the bead pellet and heated to 100°C for 10 min. After boiling, immunoprecipitates were centrifuged at 10,000 × g for 5 min, and the supernatant was collected for western blot analysis.

### Aurora-A kinase assay

The CycLex^®^ Aurora-A Kinase Assay Kit (MBL International Corporation, Woburn, MA, USA) was used to measure aurora A kinase activity in the presence of calgranulin B, following the manufacturer's instructions.

## SUPPLEMENTARY MATERIALS AND METHODS FIGURE AND TABLE




