# Supplementary material for: Antitumor effects of calgranulin B internalized in human colon cancer cells
Source: Oncotarget. 2016 Feb 27;7(15):20368–80. doi: 10.18632/oncotarget.7783 (PMC4991461; doi:10.18632/oncotarget.7783)
Supplement: Supplementary file 1 [file oncotarget-07-20368-s001.pdf]

SUPPLEMENTARY MATERIALS AND METHODS

Intravenous injection of calgranulin B in an HCT-116 cell mouse xenograft model

BALB/c-nude mice (4 weeks of age) were obtained from Japan SLC, Inc., and were group-housed (4–5 mice per cage) in plastic shoebox cages with autoclaved bedding and filtered air with access to sterilized food and water. All experimental procedures using animals were done in accordance with protocols approved by the Institutional Laboratory Animal Care and Use Committee of The Nation Cancer Center. Animals were injected with 3×10^5 HCT-116 cells in a total volume of 0.2 mL DPBS (GIBCO, New York, NY, USA). Calgranulin B was administered using intravenous injection at 10µg/kg or 50µg/kg. Tumors were measured using calipers and volumes were calculated using a standard formula: $(L \times W^2)/2$, where L = length and W = width. Differences were analyzed using the Student's t-test, and $P < 0.05$ was considered significant.

^{18}F-FDG PET/CT imaging

BALB/c-nude mice were fasted for a minimum of 6 h before PET/CT. Mice were anesthetized using 1 L/min of 2–5% isoflurane in 100% oxygen, after which they were given 740 kBq/0.1ml of ^{18}F-FDG via tail vein injection. After 20 min, ^{18}F-FDG uptake allowed for detection via animal PET/CT (GE Healthcare, Waukesha, WI, USA).

Immunohistochemistry

Tumors harvested from sacrificed mice were fixed in formalin and embedded in paraffin by routine procedures. Slides were deparafinized in xylene, rehydrated in graded alcohol and quenched in 0.3% H_2O_2 at room temperature. Slide sections were washed in distilled water, boiled in citrate buffer at 75°C, treated with protein blocking solution and washed with PBS. Immunohistochemical analyses were carried out using primary antibodies against calgranulin B (Santa Cruz Biotechnology).

SUPPLEMENTARY RESULTS

To investigate the anti-tumor effect of calgranulin B in *in vivo*, calgranulin B protein was intravenously injected into mice with implanted tumors. After calgranulin B injection, tumor volumes were determined. Both volumetric analyses (Figure S1a) and PET/CT imaging (Figure S1b) showed significant tumor volume reductions in calgranulin B-injected groups (10 µg/kg, n=5; 50 µg/kg, n=6) compared to controls (PBS only, n=5) ($P = 0.005$). In IHC analyses, calgranulin B was found only in the tumor tissues of calgranulin B-injected groups (Figure S1c). However, these results require further confirmation, because the half-life of calgranulin B is expected to be too short to cause an anti-tumor effect in an *in vivo* model. Work to modify calgranulin B without changing its specificity for colon cancer cells is ongoing.

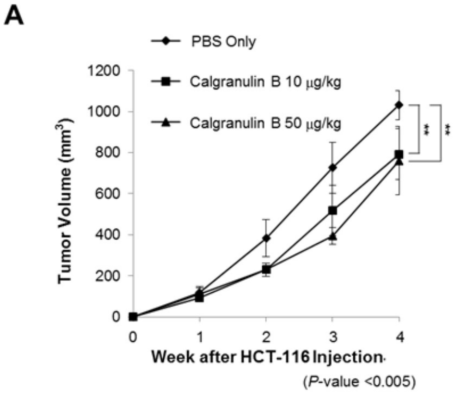

Treatment	No.	Tumor Volume (mm³) 1st Week Mass	2nd Week Mass	3rd Week Mass	4th Week Mass
PBS Only	1	87.12	289.55	541.11	922.24
	2	90.49	298.67	669.13	1001.70
	3	100.77	387.63	761.70	1055.24
	4	141.72	463.12	794.17	1085.82
	5	158.51	485.31	865.15	1093.14
Calgranulin B 10 µg/kg	1	68.63	185.11	345.67	651.78
	2	83.64	217.29	466.15	734.40
	3	88.29	230.30	526.14	754.17
	4	105.34	242.26	583.67	851.54
	5	108.29	273.21	667.32	972.66
Calgranulin B 50 µg/kg	1	79.28	193.18	332.45	587.83
	2	79.64	212.55	377.09	620.13
	3	102.87	213.39	385.60	656.26
	4	115.29	232.20	396.73	791.48
	5	131.23	249.83	431.84	925.14

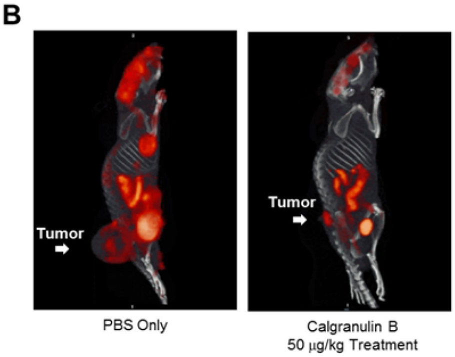

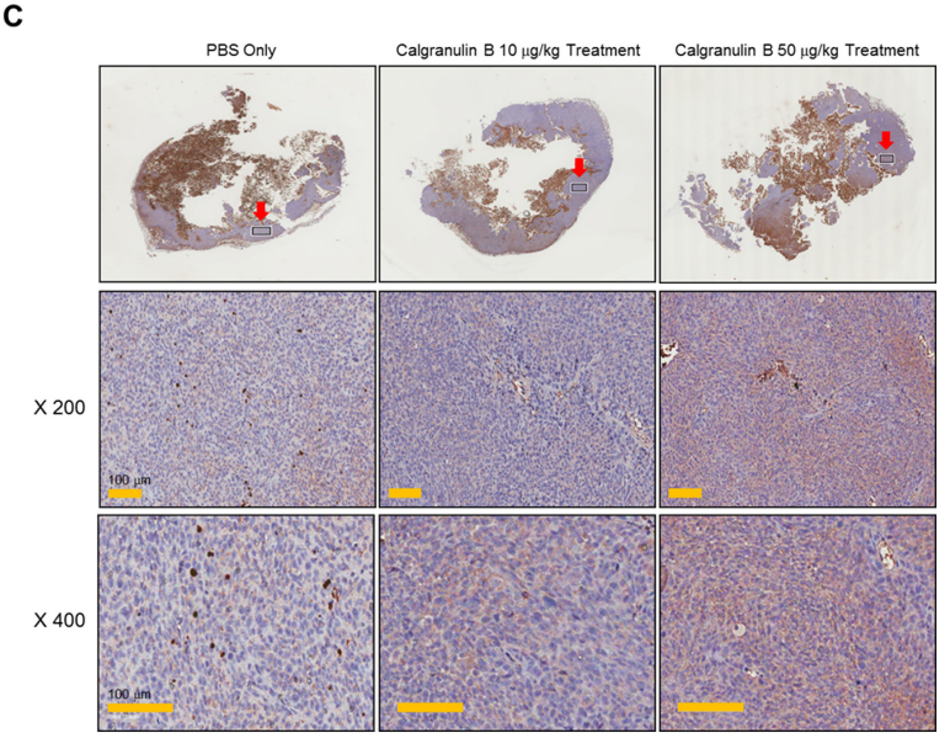

Supplementary Figure S1: Anti-tumor effect of calgranulin B in an HCT-116 colon cancer cell mouse xenograft model. **A.** Tumor volume following intravenous calgranulin B injection was significantly reduced compared to controls. **B.** ^{18}F-FDG PET/CT imaging following calgranulin B injection. **C.** IHC analysis showed that calgranulin B was found in xenografted tumor tissues obtained from calgranulin B-injected groups.

Supplementary Data 1: Identification of proteins that interact with calgranulin B using a human protein microarray.
Z-score cutoff value = 3.

See Supplementary File 1
