# Supplementary material for: Antitumor effects of calgranulin B internalized in human colon cancer cells
Source: Oncotarget. 2016 Feb 27;7(15):20368–80. doi: 10.18632/oncotarget.7783 (PMC4991461; doi:10.18632/oncotarget.7783)
Supplement: Supplementary file 2 [file oncotarget-07-20368-s002.docx]

**SUPPLEMENTARY DATA 1: Identification of proteins that interact with calgranulin B using a human protein microarray**. Z-score cutoff value = 3.

| Protein ID | Protein Name | Ultimate ORF ID | Array ID | Coefficient of Variance | Z-Score |
| --- | --- | --- | --- | --- | --- |
| NM_016619.1 | Homo sapiens placenta-specific 8 (PLAC8), | IOH10473 | B48R11C13 | 0.03834 | 15.10852 |
| NM_003621.1 | Homo sapiens PTPRF interacting protein, binding protein 2 (liprin beta 2) (PPFIBP2), | IOH10784 | B47R12C11 | 0.01983 | 10.83590 |
| BC001280.1 | Homo sapiens serine/threonine kinase 6, transcript variant 1 (aurora A kinase) | IOH21165 | B48R05C01 | 0.00805 | 10.13734 |
| NM_000993.2 | Homo sapiens ribosomal protein L31 (RPL31), | IOH14051 | B41R10C03 | 0.01729 | 10.13514 |
| NM_005435.2 | Homo sapiens Rho guanine nucleotide exchange factor (GEF) 5 (ARHGEF5), transcript variant 1 | IOH14526 | B47R03C13 | 0.06781 | 9.93084 |
| BC033621.2 | Homo sapiens pseudouridylate synthase 7 homolog (S. cerevisiae)-like | IOH21688 | B44R11C03 | 0.02472 | 9.14002 |
| BC014949.1 | Homo sapiens likely ortholog of mouse D11lgp2 | IOH13331 | B41R11C07 | 0.07971 | 8.95111 |
| NM_032345.1 | Homo sapiens within bgcn homolog (Drosophila) (WIBG) | IOH6625 | B43R09C15 | 0.01188 | 8.34042 |
| BC033758.1 | Homo sapiens centaurin, alpha 2 | IOH21879 | B47R12C05 | 0.06954 | 8.33602 |
| BC012021.1 | Homo sapiens ring finger protein 125 | IOH10818 | B48R11C15 | 0.04443 | 8.31406 |
| NM_015640.1 | Homo sapiens PAI-1 mRNA-binding protein (PAI-RBP1) | IOH22934 | B41R08C07 | 0.08258 | 8.10318 |
| NM_002070.1 | Homo sapiens guanine nucleotide binding protein, alpha inhibiting activity polypeptide 2 (GNAI2), | IOH14604 | B41R05C03 | 0.03085 | 7.87033 |
| NM_033661.1 | Homo sapiens WD repeat domain 4 (WDR4) | IOH6391 | B40R05C13 | 0.05473 | 7.54740 |
| NM_182563.2 | Homo sapiens hypothetical protein MGC21830 (MGC21830) | IOH25780 | B46R11C15 | 0.02205 | 7.46173 |
| BC007320.2 | Homo sapiens annexin A10 | IOH5857 | B47R03C13 | 0.01416 | 7.17396 |
| BC032485.1 | Homo sapiens apoptosis-inducing factor like, transcript variant 2 | IOH21724 | B42R10C15 | 0.03692 | 6.69727 |
| BC067735.1 | Homo sapiens cDNA clone MGC86968 | IOH40060 | B22R12C03 | 0.02645 | 6.17446 |
| NM_001002913.1 | Homo sapiens peptidyl-tRNA hydrolase 1 homolog (S. cerevisiae) (PTRH1) | IOH26561 | B47R10C19 | 0.04670 | 6.03386 |
| NM_016304.2 | Homo sapiens chromosome 15 open reading frame 15 (C15orf15) | IOH7552 | B47R11C17 | 0.04379 | 5.95698 |
| BC001709.1 | Homo sapiens NAD kinase | IOH4911 | B48R05C15 | 0.08107 | 5.92183 |
| BC045535.1 | Homo sapiens chromosome 1 open reading frame 25 | IOH26895 | B38R11C01 | 0.09805 | 5.76586 |
| BC016854.1 | Homo sapiens PTD015 protein | IOH10430 | B45R07C19 | 0.01789 | 5.61868 |
| BC028218.1 | Homo sapiens Z-DNA binding protein 1 | IOH11616 | B37R12C05 | 0.02596 | 5.57695 |
| NM_031412.1 | Homo sapiens GABA(A) receptor-associated protein like 1 (GABARAPL1) | IOH14808 | B38R09C01 | 0.04810 | 5.52642 |
| NM_031465.2 | Homo sapiens chromosome 12 open reading frame 32 | IOH6623 | B47R11C09 | 0.04351 | 5.49787 |
| NM_003146.2 | Homo sapiens structure specific recognition protein 1 (SSRP1) | IOH4845 | B44R12C01 | 0.07961 | 5.33311 |
| BC010501.1 | Homo sapiens catenin (cadherin-associated protein), delta 1 | IOH10253 | B40R09C17 | 0.06825 | 5.26721 |
| BC068537.1 | Homo sapiens serine palmitoyltransferase, long chain base subunit 1 | IOH40089 | B47R12C13 | 0.02209 | 5.19911 |
| BC029611.1 | Homo sapiens amyotrophic lateral sclerosis 2 (juvenile) chromosome region, candidate 4 | IOH28688 | B34R07C07 | 0.07119 | 5.16836 |
| BC007888.1 | Homo sapiens eukaryotic translation initiation factor 2, subunit 2 beta, 38kDa | IOHY29315 | B46R07C11 | 0.06572 | 5.00800 |
| BC005858.1 | Homo sapiens fibronectin 1 | IOH5967 | B45R11C17 | 0.01453 | 4.84544 |
| NM_002867.2 | Homo sapiens RAB3B, member RAS oncogene family (RAB3B) | IOH6672 | B43R05C11 | 0.06001 | 4.83886 |
| BC033856.1 | Homo sapiens La ribonucleoprotein domain family, member 1 | IOH21797 | B28R07C15 | 0.03094 | 4.83446 |
| NM_003897.2 | Homo sapiens immediate early response 3 (IER3), transcript variant short | IOH6603 | B26R10C13 | 0.05278 | 4.83446 |
| BC009762.2 | Homo sapiens tripartite motif-containing 41 | IOH14113 | B45R09C15 | 0.05610 | 4.47639 |
| NM_014868.1 | Homo sapiens ring finger protein 10 (RNF10) | IOH9787 | B41R07C05 | 0.02550 | 4.46541 |
| NM_001203.1 | Homo sapiens bone morphogenetic protein receptor, type IB (BMPR1B) |  | B25R05C13 | 0.02653 | 4.39731 |
| NM_015939.2 | Homo sapiens CGI-09 protein (CGI-09) | IOH3137 | B33R08C15 | 0.02087 | 4.32921 |
| NM_013975.1 | Homo sapiens ligase III, DNA, ATP-dependent (LIG3), transcript variant alpha | IOH40893 | B14R12C01 | 0.02318 | 4.26990 |
| NM_013375.2 | Homo sapiens activator of basal transcription 1 (ABT1) | IOH1920 | B36R09C15 | 0.07796 | 4.25672 |
| NM_005517.2 | Homo sapiens high-mobility group nucleosomal binding domain 2 (HMGN2) | IOH40568 | B26R11C09 | 0.07627 | 4.23695 |
| BC053365.1 | Homo sapiens ribosomal protein S6 kinase, 70kDa, polypeptide 1 | IOH29003 | B18R10C11 | 0.01801 | 4.19961 |
| BC007009.1 | Homo sapiens carboxypeptidase A2 (pancreatic) | IOH7191 | B48R05C03 | 0.03695 | 4.08538 |
| NM_005299.1 | Homo sapiens G protein-coupled receptor 31 (GPR31) | IOH39404 | B48R11C05 | 0.00860 | 4.05023 |
| BC006105.1 | Homo sapiens chromosome 6 open reading frame 134 | IOH5992 | B42R14C11 | 0.11435 | 4.00629 |
| NM_016271.3 | Homo sapiens ring finger protein 138 (RNF138), transcript variant 1 | IOH11194 | B47R11C13 | 0.08044 | 3.96236 |
| BC002555.1 | Homo sapiens CDC-like kinase 3, transcript variant phclk3 | IOH4008 | B38R10C01 | 0.07857 | 3.90524 |
| NM_020239.2 | Homo sapiens CDC42 small effector 1 (CDC42SE1), transcript variant 2 | IOH21482 | B48R09C09 | 0.00521 | 3.88548 |
| BC029378.1 | Homo sapiens telomeric repeat binding factor (NIMA-interacting) 1, transcript variant 2 | IOH23186 | B25R11C03 | 0.04249 | 3.88108 |
| NM_004383.1 | Homo sapiens c-src tyrosine kinase (CSK) |  | B01R05C05 | 0.10524 | 3.75807 |
| NM_000989.2 | Homo sapiens ribosomal protein L30 (RPL30) | IOH3809 | B30R09C05 | 0.01080 | 3.74628 |
| BC008730.2 | Homo sapiens hexokinase 1, transcript variant 1 | IOH5942 | B33R11C13 | 0.05613 | 3.69876 |
| BC014991.1 | Homo sapiens N-methylpurine-DNA glycosylase, transcript variant 2 | IOH12177 | B37R09C11 | 0.07194 | 3.64384 |
| NM_021639.2 | Homo sapiens GC-rich promoter binding protein 1-like 1 (GPBP1L1) | IOH10045 | B46R10C03 | 0.06849 | 3.56915 |
| NM_006433.2 | Homo sapiens granulysin (GNLY), transcript variant NKG5 | IOH27865 | B33R11C09 | 0.06077 | 3.49665 |
| BC005004.1 | Homo sapiens family with sequence similarity 64, member A | IOH4814 | B29R12C17 | 0.05013 | 3.49446 |
| BC038976.1 | Homo sapiens Rho GTPase activating protein 15 | IOH28763 | B09R06C13 | 0.03706 | 3.48567 |
| NM_033642.1 | Homo sapiens fibroblast growth factor 13 (FGF13), transcript variant 1B | IOH36760 | B35R11C17 | 0.08543 | 3.45931 |
| BC021263.1 | Homo sapiens RAB24, member RAS oncogene family, transcript variant 1 | IOH14642 | B29R04C03 | 0.14201 | 3.41099 |
| NM_021104.1 | Homo sapiens ribosomal protein L41 (RPL41), transcript variant 1 | IOH13630 | B14R10C07 | 0.04836 | 3.37584 |
| NM_006205.1 | Homo sapiens phosphodiesterase 6H, cGMP-specific | IOH40356 | B34R11C15 | 0.00255 | 3.36705 |
| NM_052845.1 | Homo sapiens methylmalonic aciduria (cobalamin deficiency) cblB type (MMAB), | IOH13544 | B45R04C03 | 0.05270 | 3.30993 |
| NM_006298.2 | Homo sapiens zinc finger protein 192 (ZNF192) | IOH34757 | B21R11C13 | 0.05283 | 3.30115 |
| BC053557.1 | Homo sapiens zinc finger protein 740 | IOH29019 | B20R09C15 | 0.02264 | 3.28138 |
| NM_138551.1 | Homo sapiens thymic stromal lymphopoietin (TSLP), transcript variant 2 | IOH13700 | B21R10C19 | 0.07185 | 3.25941 |
| NM_130398.1 | Homo sapiens exonuclease 1 (EXO1), transcript variant 2 | IOH5832 | B47R12C03 | 0.01349 | 3.16934 |
| NM_004645.1 | Homo sapiens coilin (COIL) | IOH14379 | B29R10C17 | 0.10185 | 3.16056 |
| NM_031434.2 | Homo sapiens chromosome 7 open reading frame 21 (C7orf21) | IOH3021 | B39R12C07 | 0.04527 | 3.14518 |
| NM_152736.2 | Homo sapiens zinc finger protein 187 (ZNF187), transcript variant 3 | IOH14153 | B44R10C03 | 0.02357 | 3.14079 |
| BC001132.1 | Homo sapiens DEAD (Asp-Glu-Ala-Asp) box polypeptide 54 | IOH3853 | B08R05C15 | 0.07076 | 3.09465 |
| BC016645.2 | Homo sapiens phosphoserine aminotransferase 1, transcript variant 1 | IOH25785 | B18R10C19 | 0.07991 | 3.05731 |
